# The Hap Complex in Yeasts: Structure, Assembly Mode, and Gene Regulation

**DOI:** 10.3389/fmicb.2019.01645

**Published:** 2019-07-17

**Authors:** Yinhe Mao, Changbin Chen

**Affiliations:** ^1^Key Laboratory of Molecular Virology and Immunology, Unit of Pathogenic Fungal Infection and Host Immunity, Institut Pasteur of Shanghai, Chinese Academy of Sciences, Shanghai, China; ^2^University of Chinese Academy of Sciences, Beijing, China

**Keywords:** Hap complex, CCAAT box, reactive oxygen species, iron homeostasis, yeast, cytotoxicity

## Abstract

The CCAAT box-harboring proteins represent a family of heterotrimeric transcription factors which is highly conserved in eukaryotes. In fungi, one of the particularly important homologs of this family is the Hap complex that separates the DNA-binding domain from the activation domain and imposes essential impacts on regulation of a wide range of cellular functions. So far, a comprehensive summary of this complex has been described in filamentous fungi but not in the yeast. In this review, we summarize a number of studies related to the structure and assembly mode of the Hap complex in a list of representative yeasts. Furthermore, we emphasize recent advances in understanding the regulatory functions of this complex, with a special focus on its role in regulating respiration, production of reactive oxygen species (ROS) and iron homeostasis.

## Introduction

The CCAAT box, similar as the classic TATA-box, cap signal, and GC-box, is also a major eukaryotic promoter element (Bucher, 1990). It appears in about 30% of eukaryotic genes and is located at a conserved distance of −60/−100 bp from the transcription start sites (TSS) (Maity and De Crombrugghe, 1998; Mantovani, 1999; Dolfini et al., 2009), where normally the TATA box is present (Yang et al., 2007). The CCAAT box is usually found in TATA-less promoters (Dolfini et al., 2009), and sequences around the box are highly conserved when comparing with specific genes in different species (Dorn et al., 1987; Chodosh et al., 1988a). In *S. cerevisiae*, the CCAAT box is reported to be found at promoters of the genes encoding cytochromes and those who are specifically activated by non-fermentable carbon sources (McNabb et al., 1995). In higher eukaryotes, the CCAAT box exists in all kinds of promoters but is more prevalent in cell-cycle regulated genes (Berry et al., 1992; Ronchi et al., 1996; Marziali et al., 1997; Mantovani, 1998). Deletion or mutation of this pentanucleotide dramatically affects basal levels of gene transcripts (Myers et al., 1986), highlighting its role in transcriptional regulation.

A number of DNA-binding proteins, such as the CCAAT/enhancer binding protein (C/EBP), CCAAT transcription factor/nuclear factor 1 (CTF/NF1), and CCAAT displacement protein (CDP), were found to selectively recognize the CCAAT homologous sequences (Zorbas et al., 1992; Andrés et al., 1994; Aufiero et al., 1994; Harada et al., 1995; Osada et al., 1996). Among them, nuclear factor Y (NF-Y) was originally discovered by its ability to bind to the conserved Y box element present at the promoter of the mouse MHC Class II Ea and importantly, the results, based on a series of experiments such as gel retardation, saturation mutagenesis, methylation interference, and cross-competition, clearly demonstrate that NF-Y recognition strictly requires all the five nucleotides (CCAAT) in the region and may also benefit from additional flanking sequences (Dorn et al., 1987; Karsenty et al., 1988; Ronchi et al., 1995; Mantovani, 1998). A detailed analysis in more than 100 promoters has shown that the CCAAT box and NF-Y both play important and sometimes even indispensable roles in gene regulation (Myers et al., 1986; Dorn et al., 1987; Greene et al., 1987; Karsenty et al., 1988; Danilition et al., 1991; Skalnik et al., 1991; Pramson, 1993; Szabo et al., 1993; Hasan et al., 1994; Mantovani, 1998), confirming that NF-Y is the major protein recognizing the CCAAT box (Mantovani, 1999). Actually, a list of transcription factors belonging to the NF-Y family has been discovered in a wide range of organisms and designated the Hap complex (e.g., the budding yeast *Saccharomyces cerevisiae*, the fission yeast *Schizosaccharomyces pombe*, *Kluyveromyces lactis*, *Arabidopsis thaliana*, and *Aspergillus* species) and the CBF/NF-Y (e.g., *Xenopus* and mammals), respectively (Kato, 2005). In this review, we will focus on the Hap complex subunits in yeasts and summarize recent understanding of the protein structure, function, and related signaling pathways.

## Hap Complex in Yeasts

NF-Y is a ubiquitous heteromeric protein complex composed of three subunits (NF-YA, NF-YB, and NF-YC), and is necessary for DNA binding and transcriptional regulation (Hatamochi et al., 1988; Chodosh et al., 1988a; Kim and Sheffery, 1990; Bellorini et al., 1997b). The Hap complex from *S. cerevisiae* represents the first identified factor in this family and its discovery was originated from the studies by Guarente and his colleagues. They found that this heteromeric complex contains three subunits (Hap2, Hap3, and Hap4) and was originally recognized as a transcriptional activator through its capacity of binding to the CCAAT box located in the UAS2 element of *CYC1*, a gene encoding iso-1-cytochrome C (Guarente and Ptashne, 1981; Guarente and Mason, 1983; Guarente et al., 1984; Olesen et al., 1987; Hahn and Guarente, 1988; Forsburg and Guarente, 1989; Xing et al., 2006). Interestingly, the subunit Hap4, although confers an essential activity in transcriptional activation, was found to be dispensable for the binding of the Hap complex to the CCAAT box (Olesen and Guarente, 1990; Xing et al., 1993) and a novel subunit, designated Hap5, constitutes another major component of the Hap complex and exhibits the DNA-binding capacity to the CCAAT box (McNabb et al., 1995), indicating that the domains conferring the DNA binding and activation could be different in this complex. Indeed, further studies clarified that the DNA-binding domain of the Hap complex contains three essential subunits, Hap2/3/5 (the three homologs of NF-YA, NF-YB, and NF-YC subunits), while the activation domain is composed of only Hap4 (Mantovani, 1999; McNabb and Pinto, 2005; Bolotin-Fukuhara, 2017). More importantly, gene activation in *S. cerevisiae* requires the factor Hap4, in addition to the Hap2/Hap3/Hap5 subunits (Forsburg and Guarente, 1989; McNabb et al., 1995). The Hap2/3/5 subcomplex, although constitutively expressed, needs to combine another regulatory subunit Hap4 to trigger gene expression and intriguingly, two distinct amino acid domains of Hap5 (residues 71–102 and 115–146) mediate the recruitment of Hap4 to the trimeric complex (McNabb et al., 1997).

Following characterization of the Hap complex in *S. cerevisiae*, this highly conserved factor was also discovered and analyzed in mammals including human and other vertebrates (Chodosh et al., 1988a). Three heterologous components, including NF-Y, CP1, and CBF, were found to share remarkable sequence similarities to the yeast homologs of Hap complex and studies have shown that they form a functionally conserved, multisubunit complex that is capable of binding to the CCAAT nucleotide sequences in a way similar to its ortholog in the budding yeast, although considerable divergence between yeast and mammalian complexes exists because of the varied sequences outside of the highly conserved core motifs (Chodosh et al., 1988b; Maity et al., 1990; Vuorio et al., 1990; Li et al., 1992b; Sinha et al., 1995; Becker et al., 2006; Hooft van Huijsduijnen et al., 2018). In addition to *S. cerevisiae*, homologs of the Hap complex components were also present in other yeasts such as Hap2/Hap3/Hap5 in *Candida glabrata* (Ueno et al., 2011; Thiébaut et al., 2017), Php2/Php3/Php5 in *Schizosaccharomyces pombe* (Olesen et al., 1991; McNabb et al., 1997), Hap2/Hap3/Hap5 in *Cryptococcus neoformans* (Jung et al., 2010), and Hap2/Hap3/Hap5 in *Candida albicans* (Table 1; Johnson et al., 2005; Baek et al., 2008; Hsu et al., 2011; Singh et al., 2011). Interestingly, unlike yeast and mammalian Hap complex components in which each subunit is encoded by a single gene, the Hap2/3/5 subunits in plants could be represented by multiple genes. For example, *Arabidopsis* genome harbors at least three isoforms of each of the Hap subunits and even more, searching the Plant Transcription Factor Database (PlantTFDB) identifies a total of 59 predicted genes coding for the three subunits of NF-Y in tomato, including 10 NF-YA, 29 NF-YB, and 20 NF-YC genes (Edwards et al., 2002; Siefers et al., 2008; Petroni et al., 2012; Li et al., 2016), suggesting that more diverse roles in gene transcription might be assigned to this complex in higher plants.

**Table 1:** CCAAT-box binding proteins in this paper.

Species	Name	Subunits	Reference
*Saccharomyces cerevisiae*	Hap	Hap2/3/4/5	McNabb et al., 1997; McNabb and Pinto, 2005
*Schizosaccharomyces pombe*	Php	Php2/3/4/5	Olesen et al., 1991; McNabb et al., 1997
*Cryptococcus neoformans*	Hap	Hap2/3/5/X	Jung et al., 2010
*Candida albicans*	Hap	Hap2/31/32/43/5	Johnson et al., 2005; Baek et al., 2008; Hsu et al., 2011; Singh et al., 2011
*Candida glabrata*	Hap	Hap2/3/4/5	Ueno et al., 2011; Thiébaut et al., 2017
Mammals	NF-Y	NF-YA/B/C	Hatamochi et al., 1988; Chodosh et al., 1988a; Kim and Sheffery, 1990

The Hap complex in *S. cerevisiae* binds to the CCAAT sequence in the upstream activation sequence (UAS) of numerous cytochrome genes, acting as a master regulator of respiratory metabolism (Buschlen et al., 2003; Schüller, 2003), similar to its role in the mammalian cells (Chodosh et al., 1988a). Analysis of expression profiles in *HAP2* or *HAP3* gene deletion mutant cells after a diauxic shift identified those whose expressions were significantly up- or down-regulated, and actually the CCAAT box is clearly enriched in the UAS of these genes (Bonander et al., 2008). Sequence analyses showed the presence of a conserved CCAAT box at the promoters of almost all nuclear encoded respiratory genes and strong evidence supported that activation of these genes requires the Hap2/3/4/5 complex (Buschlen et al., 2003). However, different from the mammalian cells in which the trimeric complex (NF-Y, CP1, and CBF) is sufficient to regulate gene expression, gene activation in *S. cerevisiae* requires another factor (Hap4) in addition to the Hap2/Hap3/Hap5 (Forsburg and Guarente, 1989; McNabb et al., 1995). Intriguingly, only fungi contain the Hap4 subunit (Bourgarel et al., 1999; Kato, 2005; Mercier et al., 2008). Similar to the homologs in other higher eukaryotes, Hap4 in fungi has structurally split its activation function by incorporating into NF-YA and NF-YC, the two NF-Y subunits harboring activation motifs that are displayed as long glutamine-rich stretches in the respective N-terminal (NF-YA) or C-terminal (NF-YC) regions (Li et al., 1992a; Coustry et al., 1996; Di Silvio et al., 1999; Hortschansky et al., 2017). In addition, Hap4 homologs are expressed in other yeasts whereas the function is different (Bourgarel et al., 1999; Hortschansky et al., 2007; Mercier et al., 2008; Jung et al., 2010). For example, Hap4 homologs, including the *S. pombe* homolog Php4, the *C. neoformans* homolog HapX, and the *C. albicans* homolog Hap43, were all found to act as transcriptional repressors, instead of being activators (Hsu et al., 2013). Importantly, these homologs were found to be critical in regulation and maintenance of iron homeostasis, mainly because they negatively regulate expression of iron-consuming genes under low-iron conditions.

## Architecture of the Hap Complex

NY-F has been identified in various eukaryotes, including yeasts, fungi, higher plants, and vertebrates, and it has been clear that the domains required for DNA and subunit binding in all three core subunits (NF-YA, NF-YB, and NF-YC) are evolutionarily conserved (Li et al., 1992b; Quan et al., 2018). The architecture of NY-F has been extensively studied (Mantovani, 1999; Matuoka and Yu Chen, 1999; Matuoka and Chen, 2002; Dolfini et al., 2012). With the characterization of the crystal structure of the NF-Y trimer bound to DNA (Huber et al., 2012; Nardini et al., 2013; Gnesutta et al., 2017; Nardone et al., 2017), it has been clear that the NF-YB and NF-YC interact with each other to form a tight heterodimer through a core region that belongs to the class of histone fold domain (HFD) proteins and is responsible for the non sequence-specific DNA binding. NF-YA could be divided into two domains hosting structurally extended α-helices, as the N-terminal region is required for the binding of NF-YB/NF-YC whereas the C-terminal region is involved in the recognition and binding of the CCAAT elements (Olesen and Guarente, 1990; Maity and De Crombrugghe, 1992; Xing et al., 1993, 2006; Mantovani et al., 1994; Nardini et al., 2013). In addition to the presence of the highly conserved core regions in these three subunits which are responsible for the formation of a trimer, both NF-YA and NF-YC also contain transcriptional activation domains that are glutamine rich and much less conserved (Coustry et al., 1996; Di Silvio et al., 1999). Importantly, the histone fold domain (HFD) present in the core region of NF-YB and NF-YC is not only responsible for the heterodimer interaction *via* a head-to-tail fashion that is similar to the H2A/H2B in the nucleosome (Luger et al., 1997; Romier et al., 2003) but also is needed for creating a molecular platform facilitating the binding and bending of DNA.

The architecture of the Hap complex has been extensively studied in fungi. A bifunctional *lexA-HAP2* fusion experiment revealed that the 265-amino-acid sequence of Hap2 was composed of an essential core of 65 amino acids, a signature sequence which could be further divided into a DNA recognition sequence containing 21 amino acids, although it is devoid of the classical DNA-binding motif, and a subunit association sequence containing 44 amino acids (Olesen and Guarente, 1990). In comparison, the conserved domain in Php2 of *S. pombe* is a 60-amino-acid block (Olesen et al., 1991). In comparison, the Hap3 subunit harbors a short 7-amino-acid sequence that is responsible for DNA-binding and mutations of this motif fail to recruit Hap4 to the complex and were unable to interact with Hap2 (Xing et al., 1993). Moreover, an 87-amino-acid core domain was found to be conserved in Hap5/Php5 and required for assembly of the Hap2/3/4 complex (McNabb et al., 1997). Of importance, the relative position of each binding domain within different subunits varies and the flanking region of each domain shows no apparent sequence homology between yeast and higher eukaryotes. Actually, the predictions were further supported by recent studies about the crystal structures of the Hap complex subunits (Huber et al., 2012; Nardini et al., 2013; Nardone et al., 2017). In eukaryotes, intrinsic disorders (IDs) are highly abundant, and it is also no exception for NF-YA since a holistic disorder of 96.25% was observed in a general prevalence test for detecting intrinsic disorder of transcriptional regulation (Liu et al., 2006). However, the exact functions of these disordered regions in fungal species are not well understood. Moreover, Hap2, besides of the conserved core domain, harbors a second evolutionarily conserved nuclear localization signals (NLS) which is essential for nuclear localization (Steidl et al., 2004; Tüncher et al., 2005). Apparently, this structure is not limited in yeast species, HapB, the Hap2 homologs in the filamentous fungus *Aspergillus nidulans*, also contains two putative nuclear localization signal sequences, named as NLS1 and NLS2, respectively. Interestingly, only NLS2, but not NLS1, is required for the nuclear localization of HapB (Tüncher et al., 2005).

A characteristic histone fold domain (HFD) is commonly recognized as the structural marker for all Hap3 homologs, and structurally, this motif is composed of a long central α-helix flanked by two smaller α-helices and is dimerized through the extensive hydrophobic contact in a head-to-tail assembly mode (Arents et al., 1991; Gnesutta et al., 2013; Hortschansky et al., 2017). The *C. albicans* Hap3 contains two distinct homologs, namely Hap31 and Hap32, and studies have shown that Hap31 and Hap32 individually interact with Hap2 and Hap5 to form DNA-binding complexes under different iron conditions (Singh et al., 2011; Chakravarti et al., 2017).

Hap5 was discovered through a yeast two-hybrid screen, using the core sequence of Hap2 as the bait (Singh et al., 2011). DNA mobility assays showed that no CCAAT-binding activity was detected in the *HAP5* depleting mutant, enforcing the role of Hap5 in gene activation (McNabb et al., 1995). Both *in vitro* and *in vivo* analyses of the evolutionarily conserved regions of *S. cerevisiae* Hap5 identified core domains (87 bp in the area of 154–242 amino acid residues) that are required for the assembly of the Hap2-Hap3-Hap5 heterotrimer (McNabb et al., 1995). Similarly, the core domains were also found in *S. pombe* Php5 and the mammalian homolog CBF-C. Moreover, a second, relatively smaller domain that is essential for the recruitment of Hap4 into the CCAAT-binding complex (CBC) was identified in Hap5 (115–146 amino acids) and Php5 (71–102 amino acids). However, this small domain alone is not sufficient for recruitment of Hap4 (McNabb et al., 1997).

Structural comparison of the Hap complex subunits in yeast, plant and mammalian cells suggests that the highly conserved subunits NF-YB and NF-YC were able to form a canonical HFD heterodimer that is mediated by DNA binding with the trimerization (Zemzoumi et al., 1999; Lee et al., 2003; Romier et al., 2003; Yamamoto et al., 2009; Huber et al., 2012; Nardini et al., 2013; Gnesutta et al., 2017). Nevertheless, significant differences also exist. For example, the corresponding amino acid residue that is located in helix α2 of the HFD structure and responsible for the association with the DNA phosphate backbone is very conserved and identical in both yeast and mammals, however, it differs in the plant *Arabidopsis thaliana*, as a crystal structure at 2.3 Å resolution of the core domains of the At-L1L/At-NF-YC3 dimer showed that the typical Lys/Arg present in yeast and mammalian NF-YBs was replaced with a negatively charged Asp (Asp84 in At-L1L and Asp55 in LEC1) (Gnesutta et al., 2017). This sequence diversity turns out be important for composition selectivity and sequence flexibility of GC or AT base pairs flanking the CCAAT box, and therefore reflecting functional differences in gene activation in various organisms. Moreover, a proline residue (Pro205 in *A. thaliana* NF-YA6 and Pro267 in Hap2/HapB subunit of *S. cerevisiae* and *A. nidulans*) located in A1A2 linker within the Hap/DNA complex was found to favor the correct preferred positioning of the A2 helix of the Hap2/HapB subunit toward the CCAAT box site, surprisingly, this amino acid somehow is lost in mammalian NF-YA (Nardini et al., 2013; Gnesutta et al., 2017).

Additionally, a structure-based study by Nardini et al. (2013) observed that the head-to-tail assembly of NF-YB and NF-YC subunits provides a stable and compact dimeric scaffold suitable for the interaction of NF-YA and DNA binding. Meanwhile, NF-YA was found to bind to the NF-YB/NF-YC dimer and incorporate an α helix deeply into the minor groove of DNA, and thus ensure the sequence-specific recognition to the CCAAT box. Although the crystal structures of the yeast Hap complex have yet to be solved, it is reasonable to assume the structure assembly and DNA recognition mode might be very similar, given the sequence and functional conservation of the Hap complex subunits in both yeast (Hap2/3/5) and mammals (NF-YA/NF-YB/NF-YC). This notion came from an indirect evidence showing the crystal structures of the HapB/HapC/HapE: DNA complex in the filamentous fungus *Aspergillus nidulans* (Huber et al., 2012). A sequence identity (~75%) of the histone fold motifs of HapC and HapE with their human homologs of NF-YB and NF-YC reflects a high structural conservation, as both HapC and HapE subunits adopt the similar tertiary structures and also display a nucleosome-like DNA bending by anchoring to the sugar-phosphate backbone in the minor groove of DNA (Bellorini et al., 1997a; Huber et al., 2012).

As mentioned above, Hap4 is only present in fungal species. Studies of Hap4-like protein in yeasts like *C. neoformans* (HapX) and *C. albicans* (Hap43) (Jung et al., 2010; Hsu et al., 2011) as well as in filamentous fungi, such as *Aspergillus* (HapX) and *Fusarium* (HapX) (Hortschansky et al., 2007; Schrettl et al., 2010; Lopez-Berges et al., 2012), revealed that this unique factor contains several highly conserved domains, including (1) an N-terminal CBC interaction domain; (2) a bZIP/coiled coil region mediating specific DNA binding and dimerization of basic region-leucine zipper (bZIP) transcription factors; (3) up to five cysteine-rich regions (CRR); and (4) a conserved C-terminal motif of approximate 25 amino acids (Brault et al., 2015). Similar domains also exist in the C-terminal domain of yeast bZIP regulators Yap5 and Yap7. It has been confirmed that once the stable Hap2-Hap3-Hap5 heterotrimer is formed, the highly conserved CCAAT sequence is required for the binding to Hap4 *via* the recruitment domain of Hap5 (McNabb and Pinto, 2005). In yeasts such as *S. pombe* and *C. albicans*, and also the filamentous fungus *A. nidulans*, the highly conserved 17-amino-acid HAP4-like (HAP4L) domain encompassed by the CBC interaction domain was found to contribute to interactions between the recruitment domain of Hap5 and Hap4 binding (Hortschansky et al., 2007; Singh et al., 2011; Brault et al., 2015). *S. cerevisiae* Hap4 displays a high sequence similarity to the N-terminal region of HapX; however, it lacks the bZIP domain and cysteine-rich regions (Tanaka et al., 2002). The lack of this conserved domain in CBF-C is coherent with the absence of Hap4 homologs in all mammalian complexes being analyzed so far (Mantovani, 1999).

## The Assembly and DNA-Binding Modes of Hap Complex

**Figure 1:**
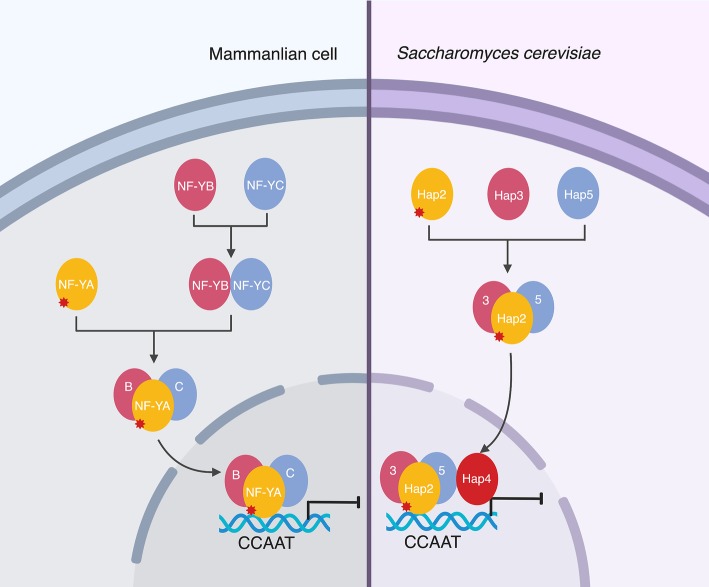
Comparison of the assembly mode of the Hap complex between yeast and mammalian cells. The symbol marked in red asterisk represents the NLS.

Although all three Hap complex subunits are required for DNA binding, only Hap2 homologs contain the nuclear localization signals (NLS). As such, an interesting question was raised: how do the other subunits transport into the nucleus? It has been generally accepted that a two-step strategy is utilized in mammalian cells. The subunits NF-YB/CBF-A and NF-YC/CBF-C form a heterodimer, afterward they interact with the third subunit, NF-YA/CBF-B, to form a heterotrimeric complex (Baxevanis et al., 1995; Sinha et al., 1995; Kato et al., 1998; Liang, 1998; Romier et al., 2003). Studies in plants like *Arabidopsis* revealed that the NF-YB/NF-YC heterodimer interacts with some specific transcription factors to form a complex (NF-YB-YC-TF) and regulates the expression of target genes through the binding of transcription factors to specific cis-elements located at the promoters (Wenkel et al., 2006; Yamamoto et al., 2009; Kumimoto et al., 2010). However, it is not the case in the yeasts. Actually, it has been found in *S. cerevisiae* that Hap2, Hap3, and Hap5 are assembled using a one-step strategy followed by the binding of Hap4 to this trimeric complex. Of importance, this recruitment only happens after Hap4 binds to the CCAAT-containing oligonucleotide (Figure 1; McNabb and Pinto, 2005). A comprehensive study using a GFP-tagged Hap subunit in both the wild-type and *hap*-deletion strains (Δ*hapB*, Δ*hapC*, and Δ*hapE*) demonstrated that the CBC is assembled in the cytoplasm first, and then the non-conserved NLS2 of Hap2 interacts with the nuclear import machinery and transports the CBC to the nucleus through a “piggy-back” mechanism (Steidl et al., 2004; Hortschansky et al., 2017).

## Gene Regulation of Hap Complex in Yeasts

**Figure 2:**
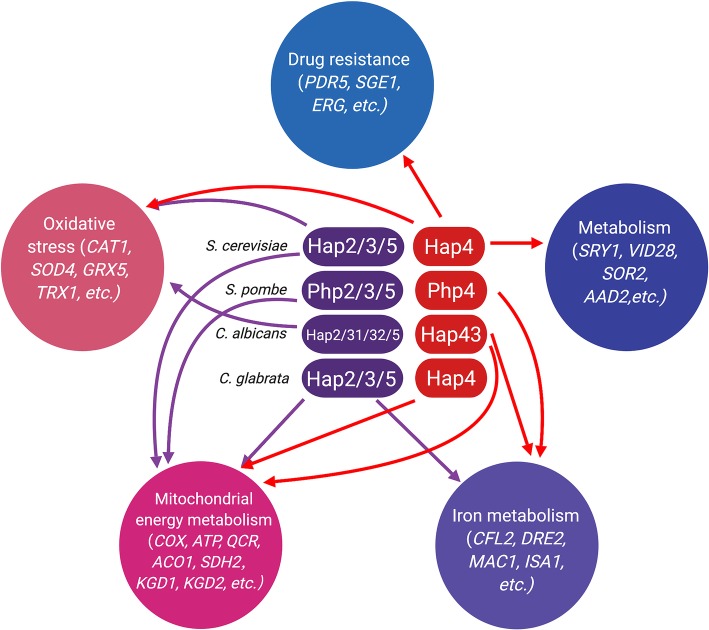
A summary of the Hap-mediated regulation in yeasts. Genes mainly regulated by the Hap2/3/5 homolog complex are marked with purple arrows, whereas genes mainly regulated by the Hap4 homolog are marked with red arrows.

Experimental data obtained from *S. cerevisiae* indicate that the Hap complex, either directly or indirectly, controls the expression of hundreds of genes, highlighting its important role as a hierarchically highly placed transactivator. In *S. cerevisiae*, approximately 230 genes are positively regulated, while approximately 240 genes are negatively regulated by the Hap complex (Figure 2; Buschlen et al., 2003). In similar, about 16% of the *C. albicans* ORFs were differentially regulated in a Hap43-dependent manner under iron-depleted conditions (Singh et al., 2011). Notably, many of the identified genes were found to play roles in sensing oxidative stress and regulating iron homeostasis. Of course, many other pathways were also found to be regulated by the Hap complex. For example, the *hap4* null mutant, in addition to its sensitivity to oxidative stress, also displayed chronological life span, decreased resistance to DNA damaging agents, smaller cell size, decreased utilization rate of nitrogen sources, etc. (Riego et al., 2002; Salmon et al., 2004; Longo et al., 2012; Bolotin-Fukuhara, 2017). In particular, the regulatory function of this complex in sensing oxidative stress and iron homeostasis will be addressed in more details.

### Respiration and Reactive Oxygen Species

The observation that the Hap complex in *S. cerevisiae* binds to the CCAAT sequence located at the UAS of almost all cytochrome genes suggests that this complex may represent a master regulator in respiratory metabolism, since these target genes belong to the mitochondrial respiratory chain (Guarente and Ptashne, 1981; Guarente and Mason, 1983; Guarente and Hoar, 1984; Buschlen et al., 2003; Schüller, 2003). Indeed, when DeRisi et al. examined the global expression patterns linked with different growth phases of *S. cerevisiae*, they found a dramatic increase of expression in genes encoding the enzymes involving TCA cycle and pathways (DeRisi et al., 1997), and intriguingly, expression of these genes was dependent on the Hap complex (De Winde and Grivell, 1993; Dang et al., 1994). This conclusion was further supported by Buschlen et al. as they also observed that the Hap complex is responsible for the upregulation of genes involved in TCA cycle (Buschlen et al., 2003). Taken together, it is clear that the Hap complex controls the complete TCA cycle and related pathways.

The mitochondrion has long been known to serve as an important compartment participating in crucial cellular events such as energy generation and metabolism in most eukaryotes. Like nucleus, mitochondrion is also a genome-bearing organelle but its genome only encodes ~1% of the total mitochondrial proteins. The majority of mitochondrial proteins is actually encoded by nuclear genes and translocated from cytoplasm to the mitochondrial compartments after translated. This phenomenon raised an important question of how nuclear and mitochondrial genomes coordinate to maintain mitochondrial biosynthesis and functionality. A growing body of studies has demonstrated a mitochondrion-nuclei communication, which is mainly governed by anterograde (nuclear to mitochondrial) or retrograde (mitochondrial to nuclear) signaling, is present under normal and pathophysiological conditions (Ng et al., 2014; Quirós et al., 2016; Guaragnella et al., 2018). Meanwhile, the mitochondrial retrograde response was firstly described in *S. cerevisiae* where the nuclear RTGs (retrograde signaling genes) were able to sense the cellular changes such as perturbed respiratory function and trigger the expression of a variety of genes involved in alteration of metabolic adaptations. One example is the glyoxylate cycle and fatty acid beta oxidation (Liu and Butow, 2006). Evidence for the integration of the Hap complex in retrograde signaling has been reported, as it has been found that the expression of *ACO1*, a mitochondrial aconitase-encoding gene in *S. cerevisiae*, is co-regulated by the Hap2/3/5 complex and the retrograde genes Rtg1 and Rtg3 (Liu and Butow, 1999; Butow and Avadhani, 2004). Deletion of both *HAP2* and *RTG1* leads to a dramatic loss of mitochondrial DNA, however, this defect could be reversed by expressing an integrated single copy of *ACO1*, suggesting that the transcriptional regulation of *ACO1* by the Hap complex and retrograde signaling pathway is able to directly affect mitochondrial DNA maintenance (Xin et al., 2005).

In addition, Buschlen et al. also found that the Hap complex regulates the expression of nuclear genes responsible for the mitochondrial processes (Galley, 2011) associated with the cellular metabolic state but not the replication and transcription of mitochondrial DNA (Buschlen et al., 2003). Interestingly, *HAP4* gene appears to be dispensable in promoting the growth rate of either aerobiosis or anaerobiosis in a standard glucose medium. However, the growth pattern is changed in a low glucose condition where the cell is still able to respire in the absence of *HAP4* gene, although its respiration capacity is significantly reduced. On the other hand, the growth rate is also related to the respiration regulation. Transcription of the respiration genes requires the Hap2/3/5 complex when the cells were grown below the intermediate specific growth rate, whereas the Hap4 protein is activated when they are above the intermediate specific growth rate (Raghevendran et al., 2006). Therefore, Hap4 plays an important role in regulating the expression of genes crucial for mitochondrial respiration and reductive pathways.

When the host is invaded by microbial pathogens, one of the major strategies that immune cells like macrophages and neutrophils act to clear the pathogen is to expose the microbe to toxic levels of reactive oxygen species (ROS) such as superoxides, peroxides, and hydroxyl radicals (Collette et al., 2014; Gazendam et al., 2014; Kaloriti et al., 2014; Dantas et al., 2015). Accordingly, in order to survive in the host environment, the pathogen has to evolve strategies to maintain the redox balance. Studies have shown that the Hap complex is one of the major redox sensors for yeast cells and a member of the fine-tuning mechanism that adapts to different levels of oxidative stress (Thön et al., 2007; Chevtzoff et al., 2010; Marinho et al., 2014). For example, the subunits Hap2, Hap31/32, and Hap5 in *C. albicans* were found to be actively involved in regulating the expression of oxidative stress genes (e.g., *CAT1*, *SOD4*, *GRX5*, and *TRX1*) in response to iron availability (Chakravarti et al., 2017).

### Iron Homeostasis

Iron is an indispensable cofactor in various cellular processes including oxygen transport, amino acid metabolism, and DNA biosynthesis. However, excessive iron may catalyze the formation of reactive oxygen species that may destroy cells by damaging lipids, proteins, and nucleic acids, through a Fenton reaction (Stohs and Bagchi, 1995; Kalinowski, 2005). In fact, in order to prevent any potential hazards caused by iron limitation or excess, cells have to precisely regulate their iron uptake and storage. Recent studies have identified the GATA-type and the bZIP-type transcription factors as key players in regulating iron homeostasis in most fungal species, and found that these factors act through a negative transcriptional feedback loop (Hortschansky et al., 2017). When the environmental iron is sufficient, iron uptake genes are directly repressed by the GATA family transcription factors. By contrast, when the environmental iron is low, the Hap complex directly represses the iron utilization genes; meanwhile, the GATA factor-encoding gene was also inhibited in order to de-repress the iron uptake genes.

In the budding yeast *S. cerevisiae*, iron regulation depends on the activation and cytoplasm-to-nucleus translocation of two transcriptional activators, Aft1 and Aft2. Under iron-depletion conditions, Aft1 and Aft2 directly bind to the conserved promoter sequences of iron uptake genes and activate the transcription, thus inducing iron acquisition from external sources and releasing free iron deposited in intracellular vacuoles (Blaiseau et al., 2001; Rutherford et al., 2002; Courel et al., 2005; Philpott and Protchenko, 2008; Yatsyshyn et al., 2009). Interestingly, Aft2 appears to behave as a weaker transcriptional activator compared to Aft1, as mutants lacking Aft1 exhibit only poor growth under iron-depletion conditions whereas mutants depleting of both Aft1 and Aft2 almost completely abolish their growth, suggesting that differential expression of iron-uptake genes might be mediated by these two factors (Rutherford et al., 2003).

Unlike the iron-regulatory mode in *S. cerevisiae*, the expression of iron-responsive genes in the basidiomycete *Ustilago maydis* (Urbs1) (Voisard et al., 2015), the ascomycetes *Penicillium chrysogenum* (Srep) (Haas et al., 1997), and *Neurospora crass* (Sre) (Zhou et al., 1998), and some pathogenic fungi like *Aspergillus fumigatus* (SreA) (Schrettl et al., 2008), *Histoplasma capsulatum* (Sre1) (Chao et al., 2008), and *Cryptococcus neoformans* (Cir1) (Won et al., 2006), is controlled by GATA-type transcriptional repressors with are characterized with the conserved Cys2/Cys2-type zinc finger domains flanked by a cysteine-rich region (Haas et al., 2008) The GATA-type repressors, through a direct binding to the conserved sequence elements of promoter, negatively regulate the expression of iron uptake genes under iron-rich conditions.

In *S. pombe*, iron homeostasis is controlled by the GATA-type transcription factor Fep1 and the CCAAT-binding factor Php4 (Bird, 2007; Labbé et al., 2007). When the environmental iron is sufficient, Fep1 binds to the GATA *cis*-acting elements present in the promoter regions of iron acquisition genes and turns off gene expression to avoid the harmful consequences of iron overload (Pelletier et al., 2002, 2003). When the environmental iron is limited, Fep1 is dissociated from DNA, leading to transcriptional de-repression of iron acquisition genes (Pelletier et al., 2002, 2007; Jbel et al., 2009). Meanwhile, the Php4-encoding gene is induced and represses transcription of the PHP complex genes. Unlike the other orthologs, Php4 lacks the b (ZIP) domain and fails to directly bind to DNA. The Php4-dependent gene induction is mediated by the association of Php4 with the monothiol glutaredoxin Grx4. In an iron-replete condition, the [2Fe-2S]^2+^ cluster coordinates the binding of Grx4 and inactivates Php4, resulting in the nuclear exportation of Php4 in a Crm1-dependent manner (Mercier and Labbé, 2009). Moreover, a spectroscopic study by Dlouhy et al. found that Grx4 was able to bind to a [2Fe-2S]^2+^ cluster in a way that is similar to previously characterized CGFS glutaredoxins (Dlouhy et al., 2017). In an iron-depleting condition, the binding of Grx4 to the [2Fe-2S]^2+^ cluster was decomposed, resulting in the functional switch of Php4 as a repressor (Brault et al., 2015; Encinar del Dedo et al., 2015). For example, a genome-wide microarray analysis showed that Php4 is responsible for the transcriptional repression of 86 genes under the condition of iron starvation (Mercier et al., 2008; Mercier and Labbé, 2009) and among them, several genes encode proteins that execute their functions in the iron-dependent metabolic pathways, such as the iron-sulfur cluster assembly, mitochondrial respiration, tricarboxylic acid cycle, and heme biosynthesis. Therefore, the strict regulation of intracellular iron levels is through the interaction between Php4 and Fep1 where each of them controls the expression of its counterpart (Mercier et al., 2008).

Analogous to *C. albicans* are Sfu1 (GATA-type factor) and Hap43 (CCAAT-binding factor). Unlike the system of *S. pombe*, the opportunistic human fungal pathogen *C. albicans* has evolved a new transcription factor (namely Sef1) that could be integrated into the widely conserved regulatory circuit containing both Sfu1 and Hap43. Sfu1 was found to inhibit the expression of *SEF1* and iron uptake genes in iron-replete conditions, however, under iron-depleting conditions, the expression of iron uptake genes and *HAP43* who is responsible for the repression of iron utilization genes was specifically induced by Sef1 (Chen et al., 2011). More importantly, deletion of the newly identified iron responsive factor Sef1 substantially attenuated the virulence of *C. albicans*, more likely due to the composite alteration of gene expression related to both iron uptake and pathogenicity (Chen et al., 2011). This could also explain a lack of *SEF1* in the non-pathogenic fungi such as *S. pombe*. In addition to Sef1, studies in the human fungal pathogen *C. albicans* also suggest that the Hap complex participates in virulence and a different mode of iron regulation might be involved (Chen et al., 2011; Hsu et al., 2011). Unlike those non-pathogenic fungi, the pathogenic fungus *C. albicans* lives in an iron-poor environment because the host sequesters most of the free iron by hemoglobin, ferritin, transferrin, and lactoferrin, thus urging the fungus to compete with the host and other microbes for the limited iron supply (Schaible and Kaufmann, 2004). By doing so, regulation of iron homeostasis by the Hap complex may evolve a different mode in *C. albicans* when compared to the nonpathogenic fungi, since the ability of this fungus to capture iron is critical for both its survival and pathogenesis (Sutak et al., 2008). Indeed, a previous study has revealed that the Hap43/Cap2 harbors an amino-terminal bipartite domain containing a fungal-specific Hap-4 like domain and a Yap-like bZIP domain and is responsible for the expression of about 16% of gene ORFs, supporting a critical role of Hap43 in regulation of iron homeostasis (Singh et al., 2011). Of noted, Hap43 directly executes its function in iron regulation. For example, a feed-forward loop exists and is required for the Hap43-dependent expression of Hap2/3/5 complex components, as Hap43 acts to maintain the mRNA level of *HAP5* under iron-depleting conditions and mutation analysis further confirmed the functional requirement of both the Hap-4 like and bZIP domains present in Hap43 (Singh et al., 2011). Remarkably, the observation that iron-triggered activation of the Hap complex components requires only Hap43, instead of the core Hap2/3/5 subunits, highly suggests the presence of additions inputs of Hap43 (Singh et al., 2011). Consistent with this hypothesis, the Hap43 regulatory cascade was found to play a dual but contrasting role (both positive and negative) in maintenance of iron homeostasis in *C. albicans*. For example, a Hap32-dependent Hap5 recruitment on promoters of Hap43-induced or repressed genes (*FRP1* and *ACO1* respectively) was identified and characterized previously (Singh et al., 2011). This robust coordination of the regulatory machinery could define a unique role of the Hap complex in iron homeostasis and pathogenicity of *C. albicans.*

Earlier studies (Chakravarti et al., 2017) established the requirement of the Hap complex in connecting the iron acquisition to oxidative stress response, by regulating the expression of genes involved in the production of ROS under iron-overloaded conditions, and the genes include the catalase-encoding gene *CAT1*, the superoxide dismutase-encoding gene *SOD4*, the glutaredoxin-encoding gene *GRX5*, and the thioredoxin-encoding gene *TRX1*. Furthermore, high levels of catalase increase resistance to oxidative stress and affect iron homeostasis by enhancing cellular demand for iron, thereby reducing the resistance to iron limitation (Pradhan et al., 2017). In addition, both iron and oxidative stress are closely linked since high iron can catalyze production of reactive oxygen species *via* the Fenton reaction (Pierre et al., 2002). Our unpublished data demonstrated that Hap43 was indeed associated with the resistance to oxidative stress when iron is replete. The dual role is also found in *Candida glabrata* (Thiébaut et al., 2017). Hap5, together with Hap4, regulates cellular respiration and contributes to iron regulation by binding to Yap5, a transcription factor containing a HAP4-like domain. Interestingly, *S. cerevisiae* lacks an ortholog of the GATA factor and alternatively evolves new iron regulators that are absent in other fungal species. Moreover, although the CBC/Hap4 complex does exist in *S. cerevisiae*, it was found that this complex acts to activate gene transcription required for respiration, instead of iron regulation (Guarente and Hoar, 1984). A phylogenetic analysis indicates that the GATA factors are lost in *Saccharomyces* species undergoing a genome-wide replication like *Saccharomyces cerevisiae* (Merhej et al., 2015). In contrast, in *Saccharomycotina* species, such as *C. albicans*, which are not concerned with whole genome duplication, iron regulation depends on typical Fep1 and Php4 orthologs (Singh et al., 2011).

Similar to the yeasts like *S. pombe*, in the filamentous fungus *A. fumigatus*, SreA (GATA-type factor) inhibits the expression of iron acquisition genes during iron adequacy, so it is important to accommodate iron excess. HapX (a CCAAT-binding factor) acts through interaction with CBC and the CBC/HapX complex is critical for adapting to both iron starvation and iron overdose. During iron starvation, the CBC/HapX complex inhibits genes expression involved in the iron-dependent pathway, such as the heme biosynthesis, to reserve iron and activate the iron carrier system to increase iron availability (Hortschansky et al., 2007; Schrettl et al., 2010). However, this complex was found to activate the expression of genes required for vacuolar iron storage and some iron-dependent signaling pathways under iron-replete conditions (Gsaller et al., 2014).

## Conclusion

In this review, we have described considerable progresses in understanding the structure and DNA-binding of the Hap complex in yeasts. Moreover, the Hap complex-dependent regulation network was evaluated. As stated, the Hap complex mainly regulates gene expression associated with the oxidative stress response, iron homeostasis, and virulence. However, our understanding of the HAP regulon is still incomplete and many important questions remain to be answered, such as specific mechanisms related to the DNA binding and iron sensing assigned by the Hap4/HapX, proteomic maps of the Hap complex and post-transcriptional/translational modification modes of Hap complex. Undoubtedly, deciphering these questions will greatly help our understanding of the Hap complex regulatory circuits in yeasts and other fungi.

## Author Contributions

YM and CC conceived and designed this review, screened and selected the articles, analyzed and interpreted the information, drafted the manuscript, and read and approved the final version of the manuscript. YM designed the figures.

### Conflict of Interest Statement

The authors declare that the research was conducted in the absence of any commercial or financial relationships that could be construed as a potential conflict of interest.
